# Supplementary figures and images for: Apoptosis resistance of senescent cells is an intrinsic barrier for senolysis induced by cardiac glycosides
Source: Cell Mol Life Sci. 2021 Oct 29;78(23):7757–76. doi: 10.1007/s00018-021-03980-x (PMC8629786; doi:10.1007/s00018-021-03980-x)

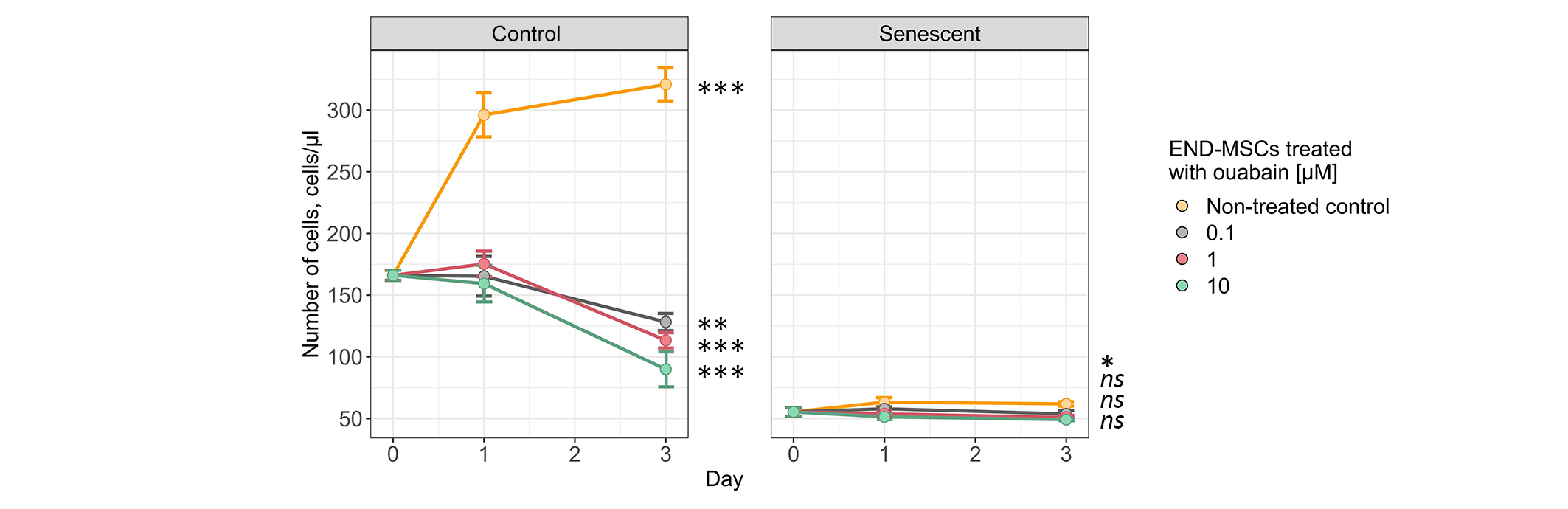

Supplement: Supplementary file 1 — Supplementary file1Supplemental Fig. S1 Standard growth curves of control and H2O2-treated senescent END-MSCs treated with ouabain. Values are mean ± SD. Statistical testing was performed using one-way ANOVA with Tukey HSD (results displayed are for Day 3 treatment outcomes against control at Day 0), n = 3, ns – not significant, * p < 0.05, ** p < 0.01, *** p < 0.001 (TIF 4320 KB) [file 18_2021_3980_MOESM1_ESM.tif]

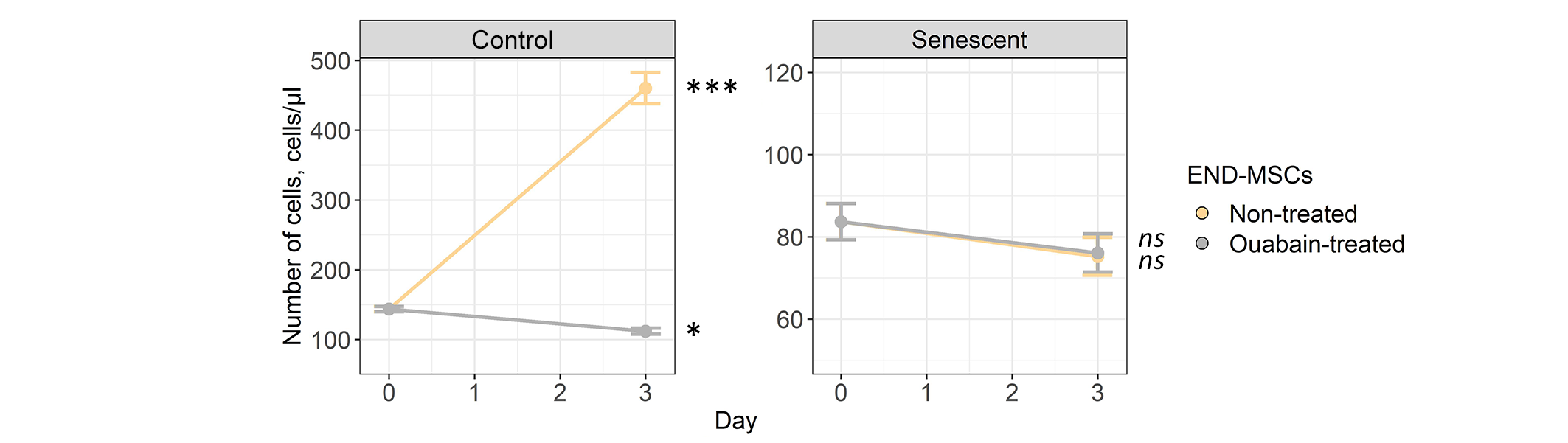

Supplement: Supplementary file 2 — Supplementary file2Supplemental Fig. S2 Growth curves of control and senescent etoposide-treated senescent END-MSCs treated with ouabain. Values are mean ± SD. Statistical testing was performed using one-way ANOVA with Tukey HSD (results displayed are for Day 3 treatment outcomes against control at Day 0), n = 3, ns – not significant, * p < 0.05, *** p < 0.001 (TIF 3765 KB) [file 18_2021_3980_MOESM2_ESM.tif]

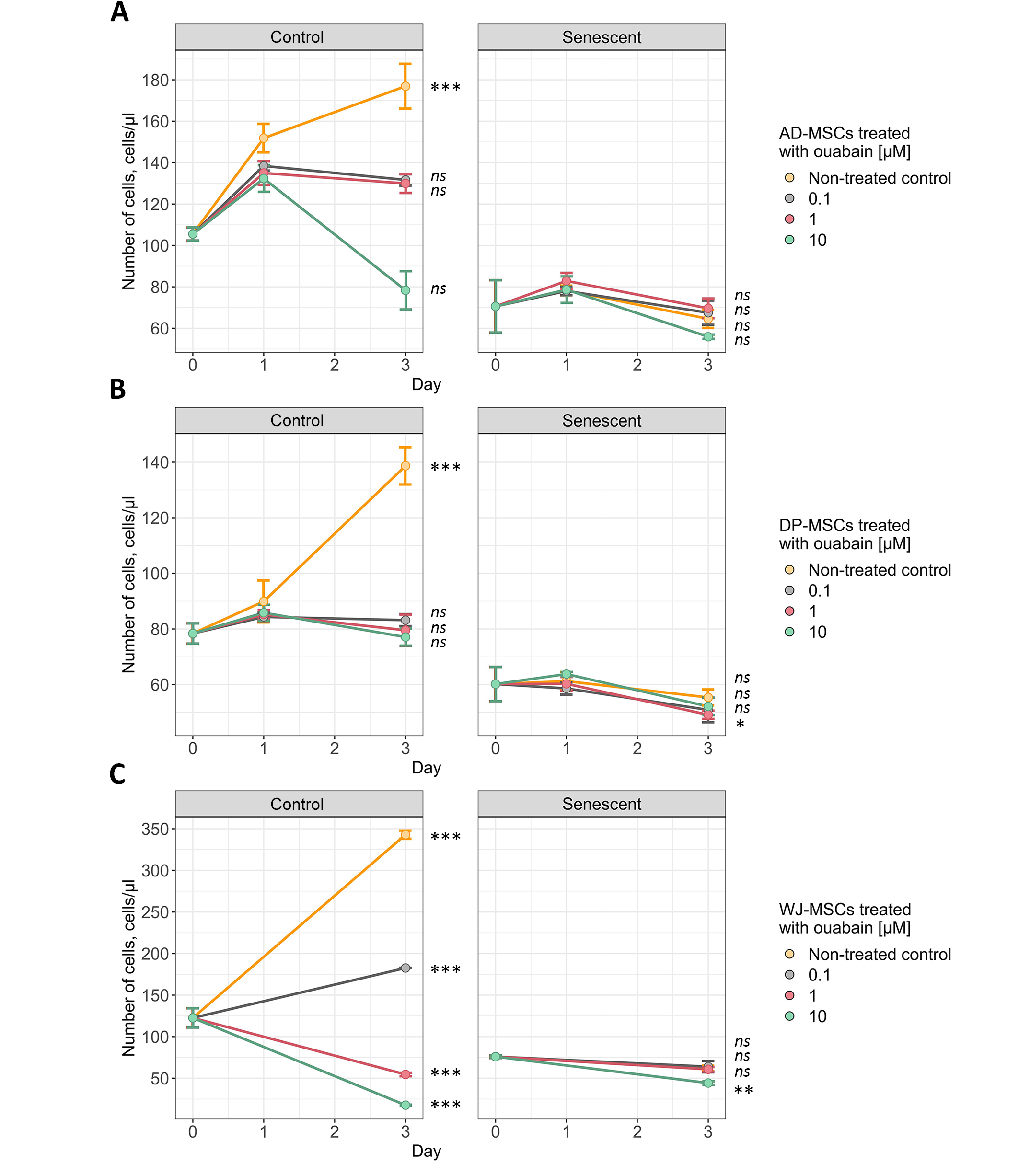

Supplement: Supplementary file 3 — Supplementary file3Supplemental Fig. S3 a, b and c Standard growth curves of control and senescent AD-MSCs, DP-MSCs and WJ-MSCs treated with ouabain, respectively. Values are mean ± SD. Statistical testing was performed using one-way ANOVA with Tukey HSD (results displayed are for Day 3 treatment outcomes against control at Day 0), n = 3, ns – not significant, * p < 0.05, *** p < 0.001 (TIF 15310 KB) [file 18_2021_3980_MOESM3_ESM.tif]

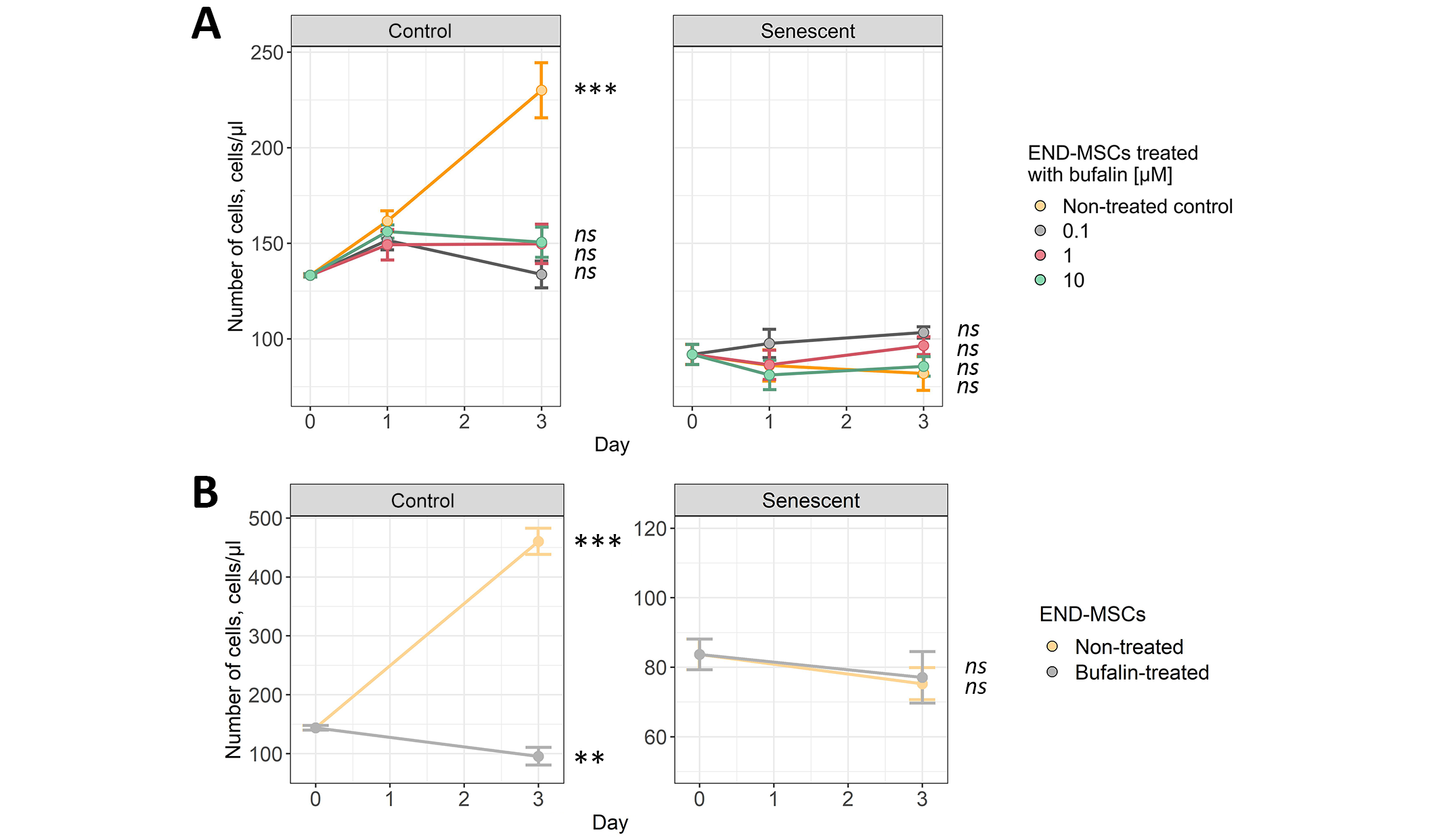

Supplement: Supplementary file 4 — Supplementary file4Supplemental Fig. S4 a, b Standard growth curves of control, H2O2-treated and etoposide-treated senescent END-MSCs supplemented with bufalin. Values are mean ± SD. Statistical testing was performed using one-way ANOVA with Tukey HSD (results displayed are for Day 3 treatment outcomes against control at Day 0), n = 3, ns – not significant, * p < 0.05, *** p < 0.001 (TIF 7753 KB) [file 18_2021_3980_MOESM4_ESM.tif]

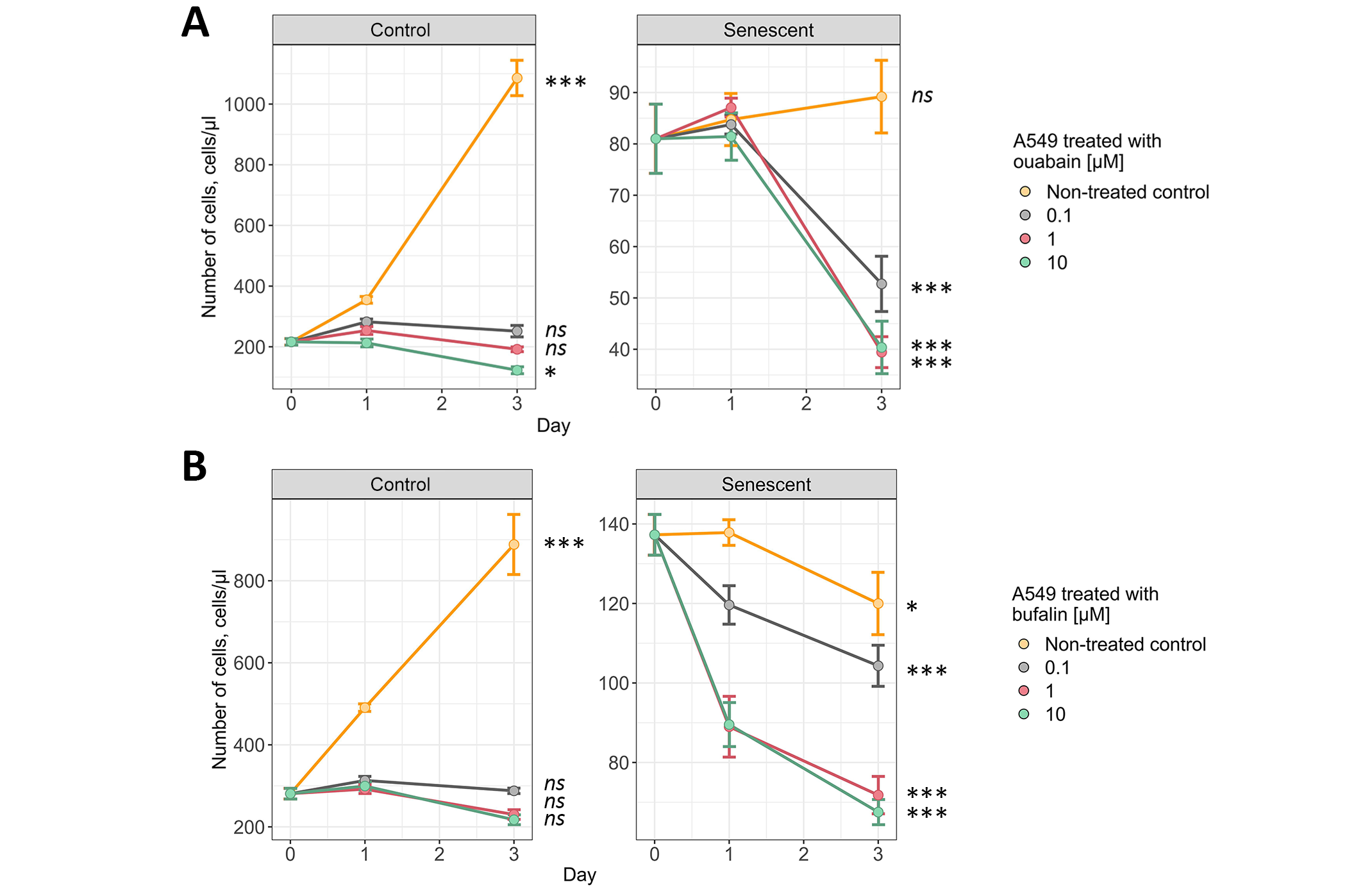

Supplement: Supplementary file 5 — Supplementary file5Supplemental Fig. S5 a, b Growth curves of control and senescent A549 cells treated with ouabain/bufalin. Values are mean ± SD. Statistical testing was performed using one-way ANOVA with Tukey HSD (results displayed are for Day 3 treatment outcomes against control at Day 0), n = 3, ns – not significant, *** p < 0.001 (TIF 8731 KB) [file 18_2021_3980_MOESM5_ESM.tif]

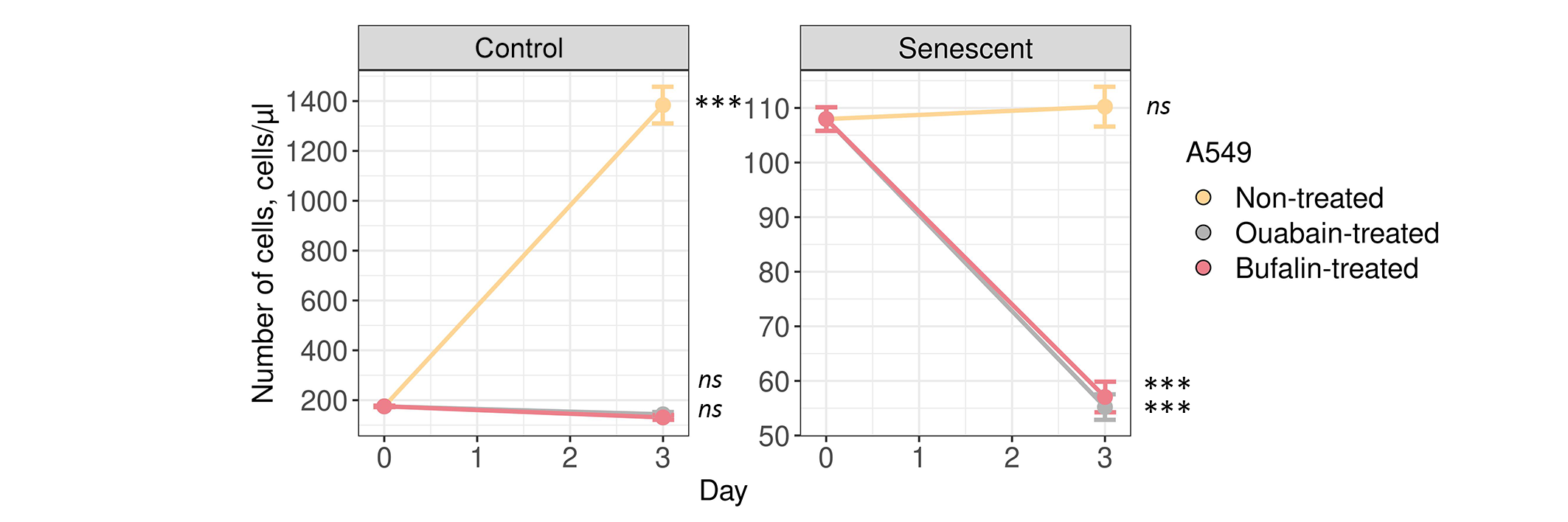

Supplement: Supplementary file 6 — Supplementary file6Supplemental Fig. S6 Standard growth curves of control and doxorubicin-treated senescent A549 treated with ouabain or bufalin, respectively. Values are mean ± SD. Statistical testing was performed using one-way ANOVA with Tukey HSD (results displayed are for Day 3 treatment outcomes against control at Day 0), n = 3, ns – not significant, * p < 0.05, *** p < 0.001 (TIF 4432 KB) [file 18_2021_3980_MOESM6_ESM.tif]

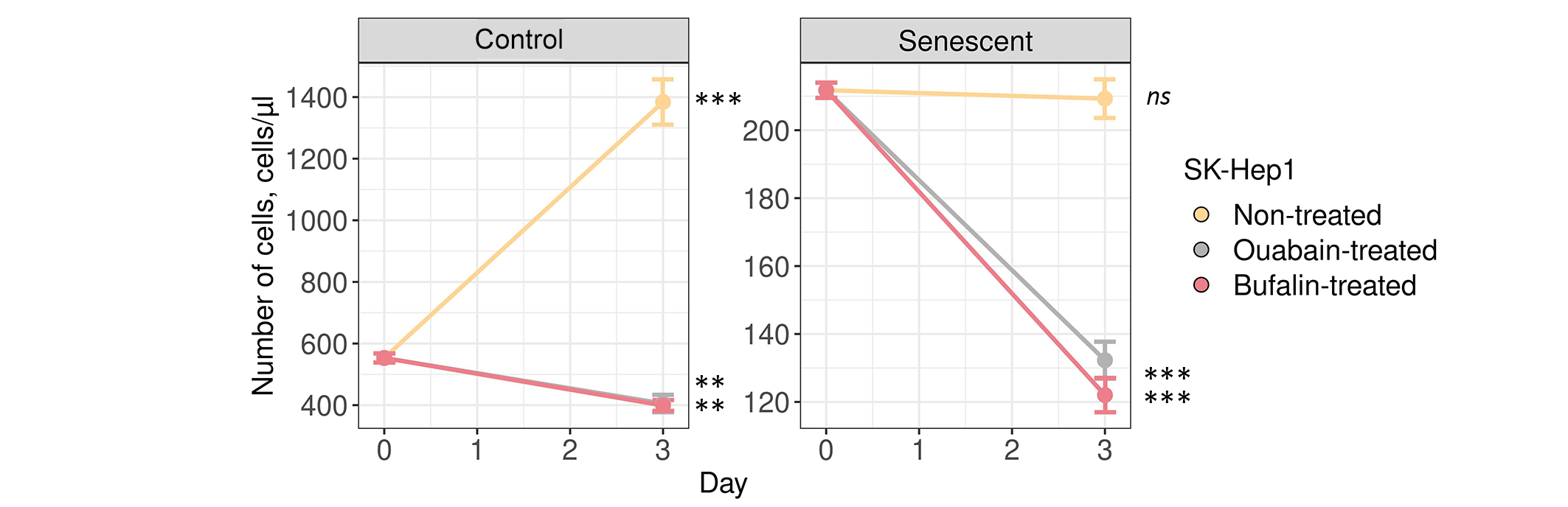

Supplement: Supplementary file 7 — Supplementary file7Supplemental Fig. S7 Standard growth curves of control and etoposide-treated senescent SK-Hep1 treated with ouabain or bufalin, respectively. Values are mean ± SD. Statistical testing was performed using one-way ANOVA with Tukey HSD (results displayed are for Day 3 treatment outcomes against control at Day 0), n = 3, ns – not significant, * p < 0.05, *** p < 0.001 (TIF 4308 KB) [file 18_2021_3980_MOESM7_ESM.tif]

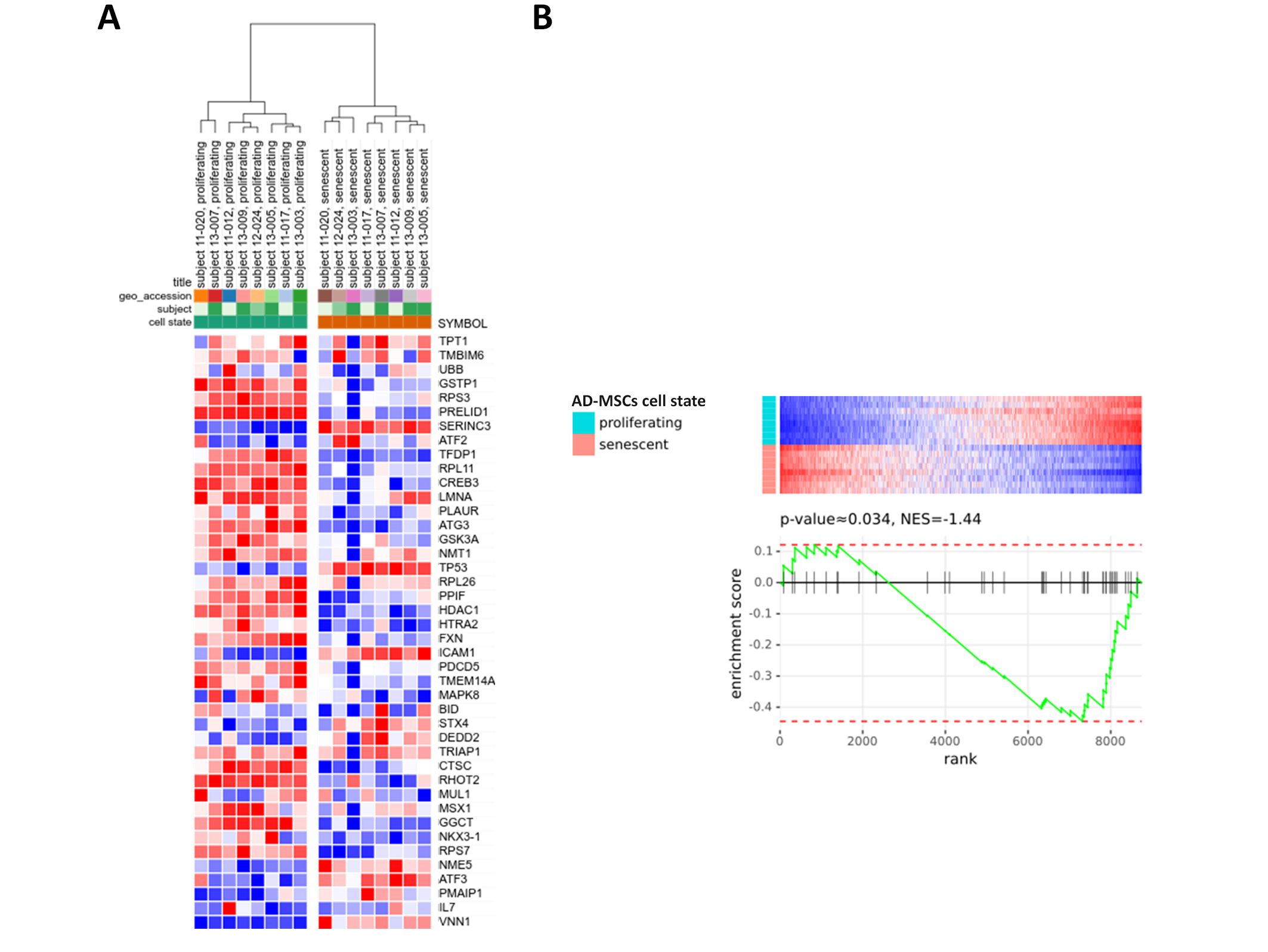

Supplement: Supplementary file 8 — Supplementary file8Supplemental Fig. S8 Senescence of ouabain-resistant AD-MSCs is accompanied by acquisition of apoptosis-resistant phenotype a Heatmap reflecting expression of the apoptosis-related genes from Fig. 11. b GSEA results for the DEGs between senescent vs control AD-MSCs for the corresponding set of genes from a (TIF 9966 KB) [file 18_2021_3980_MOESM8_ESM.tif]

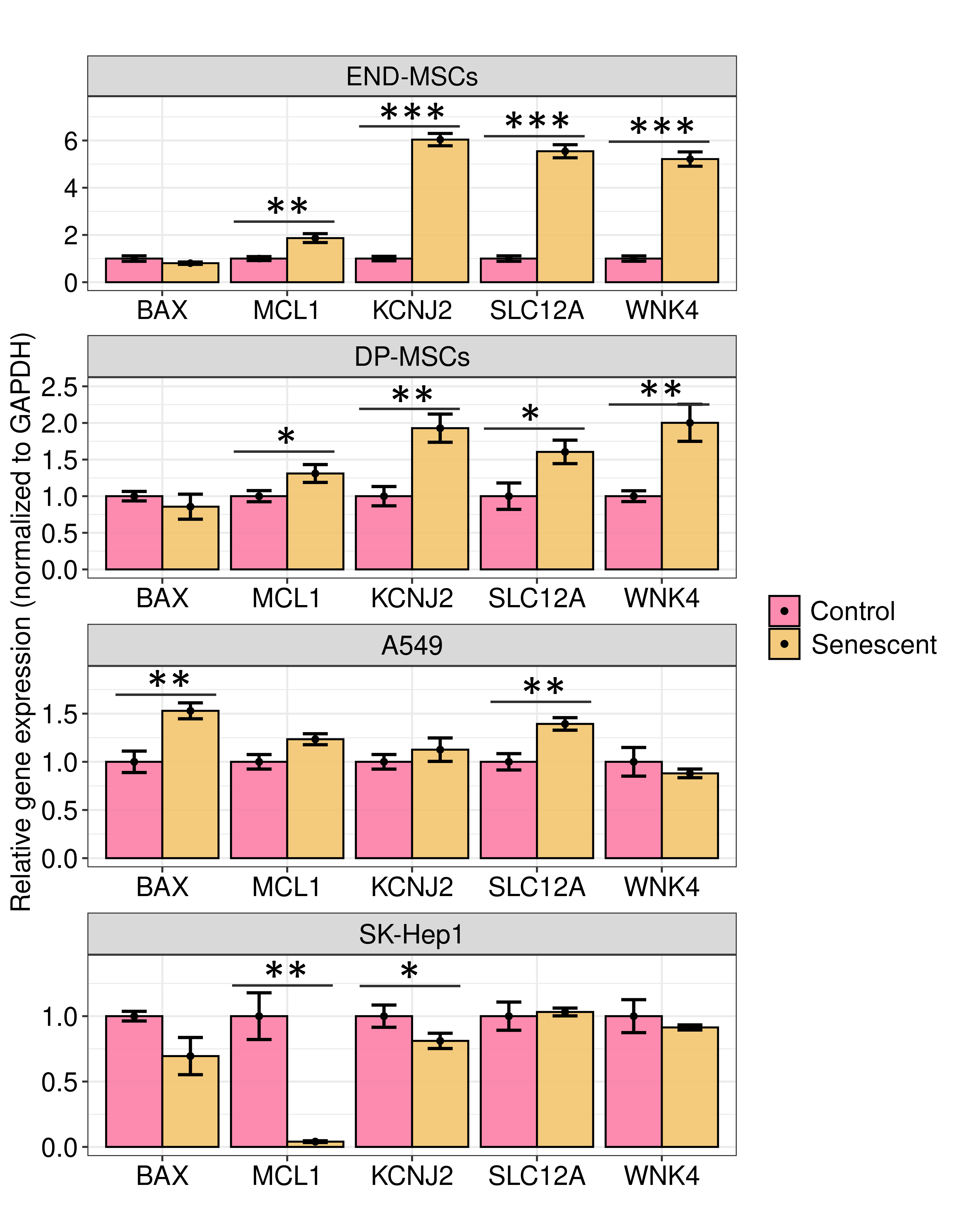

Supplement: Supplementary file 9 — Supplementary file9Supplemental Fig. S9 Analysis of BAX, KCNJ2, MCL1, SLC12A and WNK4 genes expression in control and senescent cells using the following models: oxidative stress-induced senescence of END-MSCs, etoposide-induced senescence of A549, doxorubicin-induced senescence of DP-MSCs and etoposide-induced senescence of SK-Hep1. Values are mean ± SD. Statistical significance was assessed by the Student's t test: *p < 0.05, **p < 0.01, ***p < 0.001, ns – not significant (TIF 46167 KB) [file 18_2021_3980_MOESM9_ESM.tif]
